# Pharmacophore-Based Virtual Screening of Novel Competitive Inhibitors of the Neurodegenerative Disease Target Kynurenine-3-Monooxygenase

**DOI:** 10.3390/molecules26113314

**Published:** 2021-05-31

**Authors:** Lizaveta Gotina, Seon Hee Seo, Chae Won Kim, Sang Min Lim, Ae Nim Pae

**Affiliations:** 1 Division of Bio-Medical Science & Technology, KIST School, Korea University of Science and Technology (UST), Seoul 02792, Korea; liz.chem.13@kist.re.kr (L.G.); smlim28@kist.re.kr (S.M.L.); 2 Convergence Research Center for Diagnosis, Treatment and Care System of Dementia, Korea Institute of Science and Technology (KIST), Seoul 02792, Korea; shseo@kist.re.kr (S.H.S.); Kchaewon36@naver.com (C.W.K.)

**Keywords:** kynurenine monooxygenase, flavoprotein hydroxylase, virtual screening, pharmacophore, structure-based drug design, hydrogen peroxide, Alzheimer’s disease, neurodegeneration

## Abstract

The pathogenesis of several neurodegenerative diseases such as Alzheimer’s or Huntington’s disease has been associated with metabolic dysfunctions caused by imbalances in the brain and cerebral spinal fluid levels of neuroactive metabolites. Kynurenine monooxygenase (KMO) is considered an ideal therapeutic target for the regulation of neuroactive tryptophan metabolites. Despite significant efforts, the known KMO inhibitors lack blood–brain barrier (BBB) permeability and upon the mimicking of the substrate binding mode, are subject to produce reactive oxygen species as a side reaction. The computational drug design is further complicated by the absence of complete crystal structure information for human KMO (hKMO). In the current work, we performed virtual screening of readily available compounds using several protein–ligand complex pharmacophores. Each of the pharmacophores accounts for one of three distinct reported KMO protein-inhibitor binding conformations. As a result, six novel KMO inhibitors were discovered based on an in vitro fluorescence assay. Compounds VS1 and VS6 were predicted to be BBB permeable and avoid the hydrogen peroxide production dilemma, making them valuable, novel hit compounds for further drug property optimization and advancement in the drug design pipeline.

## 1. Introduction

**Figure 1:**
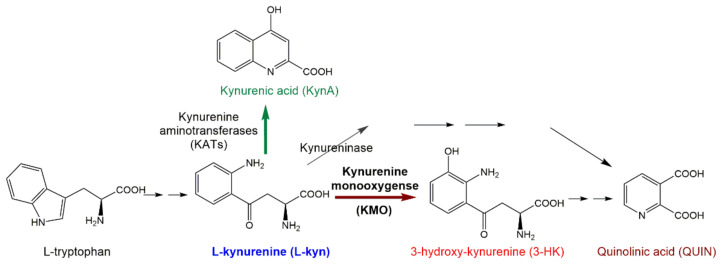
Simplified scheme of the kynurenine pathway of tryptophan metabolism. Neuroprotective metabolites are labeled in green color, neurotoxic in red.

Kynurenine-3-monooxygenase (KMO, in the past also referred to as kynurenine-3-hydroxylase), has emerged as a therapeutic target for the treatment of numerous neurological disorders including Alzheimer’s disease (AD) [1,2,3], Huntington’s disease (HD) [4,5], Parkinson’s disease (PD) [6], schizophrenia [7,8,9], depression [10,11], and neuropathic pain [12,13,14] and its inhibition has been found to be protective against cancer [15] and multiple organ disorder in acute pancreatitis [16]. Located at a critical branch point in the main metabolic pathway of L-tryptophan, KMO facilitates the hydroxylation of L-kynurenine (L-kyn) to 3-hydroxy-kynurenine (3-HK) as opposed to the kynurenine aminotransferase (KAT) branch, which catalyzes the cyclization of L-kyn to form kynurenic acid (KynA) (Figure 1). For neurological disorders, the positive effect of KMO inhibition is associated with reducing elevated levels of the downstream metabolites 3-HK and quinolinic acid (QUIN), both of which are highly reactive free radicals, and the latter is well-known to induce excitotoxicity in neurons such as NMDAR and AMPAR receptor agonists [17].

**Figure 2:**
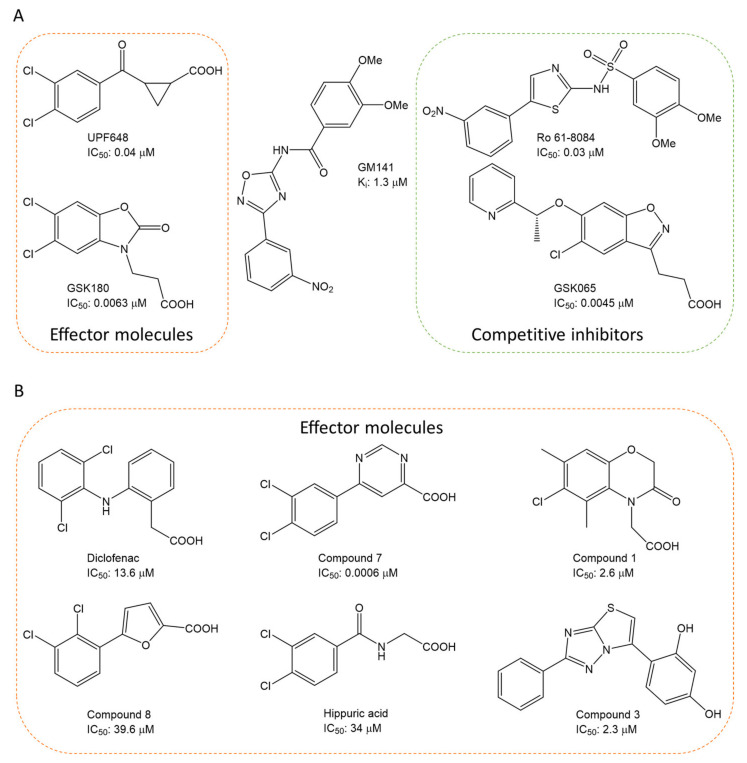
Chemical structures and potency of known KMO inhibitors. (**A**) Compounds discovered by applying substrate-based rational design. (**B**) Novel inhibitors discovered using molecular modeling approaches.

Over the past 25 years, several classes of novel and highly potent KMO inhibitors (sulfonamides [18,19], aryl-pyriminides [20], benzoylalanin derivatives [21,22], oxazolidinones [23,24]) have been discovered through substrate-based rational design approaches (Figure 2A). Independent in vivo studies performed with several promising KMO inhibitors UPF 648 [25], Ro 61-8048 [26], and 4,4-dichlorobenzoylalanine [27] have demonstrated a positive therapeutic effect of decreasing the QUIN/KynA ratio in the brain, mainly via an increase of KynA levels. However, the absolute majority of current KMO inhibitors contain a carboxylic acid group or are considered BBB impermeable; hence they have a very limited effect on reducing the elevated levels of neurotoxic QUIN in the brain. Therefore, it has been suggested that the BBB permeability of KMO inhibitors should be enhanced to achieve both therapeutic benefits of increased levels of KynA and decreased levels of downstream neurotoxic metabolites 3-HK and QUIN for the treatment of CNS disorders [28,29]. Modest success in identifying a BBB permeable KMO inhibitor was first achieved only very recently via a prodrug strategy [30], whereas the first BBB permeable KMO inhibitor had just been reported upon the writing of the current article [31].

Another substantial disadvantage of known substrate-like KMO inhibitors is that they act as non-substrate effector molecules (Type I inhibitors) by stimulating the flavin reduction by NADPH and, as a consequence, generate cytotoxic hydrogen peroxide [32]. It has been reported that upon binding of L-kyn or non-substrate effectors, a conformational change occurs in the active site, which would facilitate the NADPH cofactor binding and hydride transfer [33]. Afterward, quick O_2_ binding produces a highly reactive hydroxyflavin intermediate, which, in the absence of a hydroxable position and proper solvent shielding, decomposes, producing hydrogen peroxide. This undesirable and potentially toxic side reaction is known as the oxygen dilemma [34], and inhibitors that can overcome it have been named as competitive inhibitors (Type II inhibitors). Crystallographic and biochemical studies performed on human KMO (hKMO) and its *Saccharomyces cerevisiae* (ScKMO) and *Pseudomonas fluorescens* (PfKMO) homologues have proposed two possible approaches to designing competitive inhibitors. In the first approach, Kim et al. reported two distinct binding modes of the Ro 61-8084 inhibitor (PDB ID: 5x6r, 5x6q) [33]. Ro 61-8084 acquired a non-substrate effector binding mode in PfKMO, however, in ScKMO and hKMO, it acted as a competitive inhibitor. It was proposed that Ro 61-8084 is capable of binding to the KMO apo conformation, thus not causing any conformational changes that would stimulate NADPH binding [33]. In an alternative approach, benzisoxazole compounds were found to capture the FAD cofactor in a “tilted”, unproductive conformation (PDB ID: 5nae, 5nag, 5nah) and thereby prevent either NADPH binding or the hydride transfer reaction [35].

The crystal structure of hKMO has been reported only in an auto-inhibited conformation, unsuitable for structure-based drug design (PDB id: 5x68) [33]. Nevertheless, both ligand-based (similarity search [36], multiple-QSAR [37]) and structure-based (pharmacophore-based virtual screening [30,38] and docking-based virtual screening [39]) computational modeling approaches have been exploited to discover novel KMO inhibitors (Figure 2B). However, in all of the mentioned studies, the pharmacophores, active compounds, or KMO crystal structures used to create the models were based on L-kyn or potent non-substrate effector compounds. As a result, the discovered novel inhibitors lacked structural diversity compared to previously reported inhibitors, shared too much structural similarity with non-substrate effectors, and almost always contained a carboxylic acid moiety, thereby limiting the BBB permeability.

Herein, we address the disadvantages of the previous studies and aim to discover novel, structurally diverse competitive KMO inhibitors that would overcome the oxygen dilemma and be BBB permeable. Three crystal structures of ScKMO and PfKMO were chosen to represent all of the known binding modes of KMO inhibitors: two competitive inhibitor (Type II, not producing H_2_O_2_) structures and one non-substrate effector (Type I, substrate-like binding) structure. Three separate hKMO homology models were created to account for the structural and amino acid sequence differences, and used in a protein-ligand complex-based pharmacophore virtual screening approach to identify novel compounds.

## 2. Results

### 2.1. Homology Modeling and Structure Validation

The amino acid sequences of ScKMO and PfKMO respectively shared 35.3% and 33.7% identity and 59.6% and 56.7% similarity to hKMO. Although the overall sequence similarity was not high, the active site was mostly conserved among all three proteins, with only minor differences within 6Å of the co-crystallized ligands (Figure S1). The most notable difference was the Phe313 residue of a loop above the isoalloxazine ring system of FAD, which was replaced by Tyr323 in ScKMO or His320 in PfKMO. To best represent hKMO and consider the protein sequence differences, we decided to apply homology modeling. The following three homology models were created, each accounting for a different ligand binding mode:

- Model 1: Competitive inhibitor model (Type II), which represents the Ro 61-8084 binding mode in ScKMO. As mentioned previously, Ro 61-8084 inhibits KMO without producing H_2_O_2_ in ScKMO and hKMO.
- Model 2: Non-substrate effector model (Type I) created based on a high-resolution crystal structure of PfKMO bound to the native substrate L-kyn.
- Model 3: Competitive inhibitor model (Type II) based on PfKMO, in which FAD is trapped in an unproductive, tilted conformation. This inhibitor binding mode is also reported not to cause H_2_O_2_ production.

The 3D protein structure validation methods showed all models to be of high quality and comparable to the initial structural templates’ quality, as summarized in Table 1. The RMSD to the template structures was within 1 Å, indicating close amino acid chain positions to the initial templates. Over 90% of residues had a valid 3D-Profiles score, indicating a correct environment (secondary structure, polarity, buried surface) close to each residue. The total 3D profile score was close to the expected high score. The backbone dihedral angles of 95% of non-glycine, non-proline residues were in the energetically allowed region of the Ramachandran plot. All invalid residues were further than 15 Å away from the active site, hence they were considered insignificant discrepancies, which would have little effect on the quality of further docking studies and virtual screening. Finally, the protein reliability report confirmed the high quality of the models, indicating only slight deviations in peptide planarity and buried donor and acceptor atom surfaces (Figure S2).

### 2.2. Reference Inhibitor Docking Studies

In addition to the protein structure validation, the capability of the prepared homology models to reproduce known KMO inhibitor binding modes and correctly score them was investigated. In all three homology models, the co-crystal ligand binding poses from 11 different KMO inhibitors were reproduced accurately—within 1 Å heavy atom RMSD or less (Table S2). We expanded the inhibitor validation set to include not only highly active co-crystallized compounds, but also their moderately active analogues as well as known highly active inhibitors for which there are no reported crystal structures. The predicted binding modes for this enlarged validation set compounds were in line with the crystal structure poses, and the -CDOCKER docking score adequately represented the change in inhibitor activity, with correlation coefficients R^2^ more than 0.8 for all three models (Figure S3). Overall, the performed docking study confirmed the ability of the homology models and docking protocols to produce correct ligand binding modes with docking scores that correspond to the inhibitor activity.

### 2.3. Pharmacophore Generation

**Figure 3:**
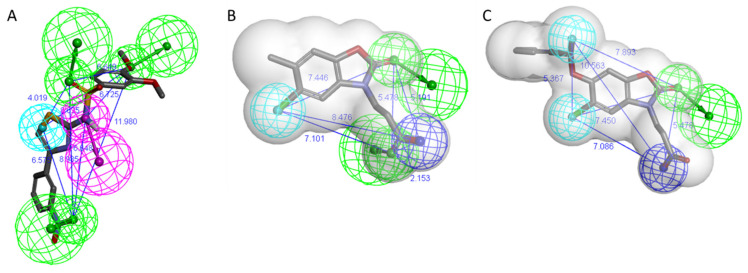
Protein–ligand complex-based pharmacophores. (**A**) Ro 61-8084 based (Model 1); (**B**) GSK428 based (Model 2); (**C**) GSK775 based (Model 3). Features are drawn as spheres (green—hydrogen band acceptor, magenta—hydrogen bond donor, sky blue—hydrophobic, dark blue—negative ionizable), the distance between features (Å) is labeled in blue, shape feature is represented as a grey transparent surface.

For virtual screening, protein–ligand complex-based pharmacophores were generated. The docking poses of KMO inhibitors Ro 61-8084, GSK428, and GSK775 were chosen to represent the inhibitor binding mode in Models 1–3, respectively. For each protein–ligand complex, 10 pharmacophore models were created and subjected to an enrichment and decoy molecule recognition test (refer to Methods section for details). The best models exhibited a strong ability to distinguish active inhibitors from the decoy molecules: the sensitivity and specificity for all three models was over 0.85, as shown in Table 2. All three models included five features (Figure 3). Notably, it was necessary to add a structure shape feature to the pharmacophores of Models 2 and 3 in order to obtain high specificity, whereas the pharmacophore for Model 1 did not require such a feature to pass the validation tests successfully.

Respectively, the pharmacophore model’s receiver operating characteristic (ROC) curves predicted excellent model quality (Figure S4): the area under the curve was ~0.9 or higher for all three models, indicating a substantial predictive ability in comparison to random guessing.

### 2.4. Virtual Screening

**Figure 4:**
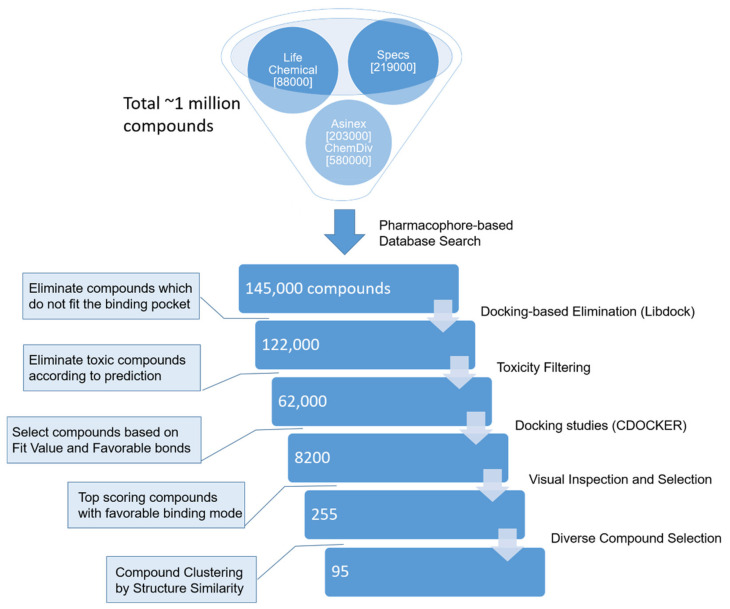
General overview of the virtual screening workflow.

In the designed virtual screening workflow, both the validated protein–ligand complex pharmacophores and molecular docking protocols were applied to screen a composite library of available on-demand compounds (Figure 4). The screening was performed for each pharmacophore and corresponding KMO homology model separately. As shown in Table 3, the pharmacophore screening based on Models 2 and 3 produced a significantly smaller number of compounds compared to Model 1, which is probably due to the presence of the shape feature in the former, which limited the size of the hit molecules. For the same reason, more than 95% of compounds detected by Models 2 and 3 pharmacophores passed the libdock screening, whereas 17% of Model 1 compounds failed to produce a preliminary docking pose.

After the toxicology filtering, the number of Model 1 compounds remained too large to directly perform molecular docking within reasonable time scales; therefore, 6925 compounds with the highest pharmacophore fit value and number of favorable protein–ligand interactions in the preliminary libdock pose were selected. The final compound selection was based on the compounds’ CDOCKER docking score and the presence of favorable interactions with important KMO residues. As a result, 64% of the final selection compounds were from the Model 1 screening, and the least-represented were Model 3 compounds (16%).

The 255 final selection compounds were grouped according to their structural similarity, and the best-performing compounds from each cluster were included in the final, diverse compound list for biological screening.

### 2.5. Identified Novel KMO Inhibitors

As a result of the virtual screening, 95 compounds were purchased from the corresponding chemical vendors and subjected to in vitro hKMO enzyme inhibition activity measurements via the KMO fluorescence-based assay. The known competitive inhibitor Ro 61-8084 served as the positive control for the assay setup validation. In the obtained dose-response curve, the Ro 61-8084 IC_50_ value was equal to 0.727 ± 0.088 µM (Figure S5), which is in agreement with previously reported inhibitor activity [31,40,41]. The compound screening resulted in identifying six novel KMO inhibitors with IC_50_ ≤ 10 µM (Table 4 and Table S4). After discovering the hit compounds, they were docked into all three hKMO models, according to the setup docking protocol, to see which binding modes appear to be more favorable.

**Figure 5:**
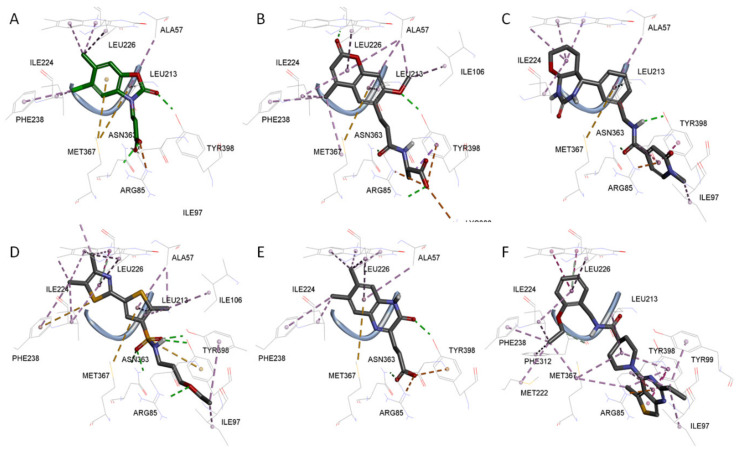
3D docking poses of compounds GSK428 (**A**), VS2 (**B**), VS3 (**C**), VS4 (**D**), VS5 (**E**), VS6 (**F**) in Model 2. Docked compounds are shown in stick format, the interacting residues and FAD cofactor are drawn in line representation. Favorable interactions shown as dashed lines: green—hydrogen bonds, yellow—π–sulfur, orange—π–charge, dark pink—π–π stacking interactions, purple—π–sigma interactions, light pink—hydrophobic interactions.

**Figure 6:**
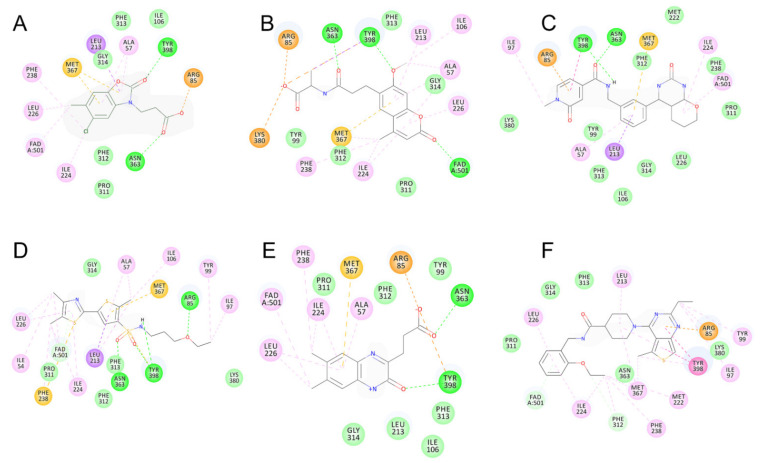
2D interaction patterns of compounds GSK428 (**A**), VS2 (**B**), VS3 (**C**), VS4 (**D**), VS5 (**E**), VS6 (**F**) in Model 2. Favorable interactions are color coded as follows: green—hydrogen bonds, yellow—π–sulfur, orange—π–charge, dark pink—π–π stacking interactions, purple—π–sigma interactions, light pink—hydrophobic interactions. Residues shown in light green form weak van der Waals interactions.

Among the discovered hit compounds, VS2 and VS5 contain a carboxylic acid moiety and are structurally similar to previously reported KMO inhibitors by Zhang et al. [30] (compounds **1** and **8**). Four out of six hit compounds contain an amide moiety, whereas VS4 is a sulfonamide compound. The docking poses of compounds VS2 and VS5 have a strong resemblance with non-substrate effector compounds (Figure 5B,E): the carboxylate moiety forms hydrogen bonds and attractive charge interactions with Arg85 and Asn363 as well as π–charge interactions with Tyr398. The methoxy oxygen of VS2 and the carbonyl oxygen of VS5 form a hydrogen bond with Tyr 398. Finally, the compounds’ fused core exhibits π–sulfur interactions with Met367 and hydrophilic interactions with Ala57, Ile206, Leu224, and Leu226, which are known to comprise the hydrophobic KMO binding pocket region. Together, these interactions are also present in the binding pattern of the initial pharmacophore-modeling compound GSK428 (Figure 6).

**Figure 7:**
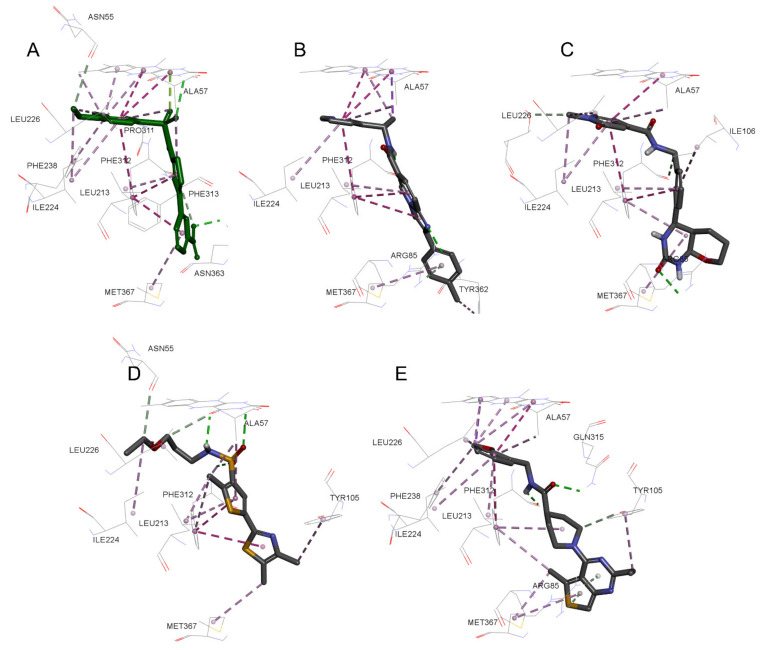
3D docking poses of compounds Ro 61-8084 (**A**), VS1 (**B**), VS3 (**C**), VS4 (**D**), VS6 (**E**) in Model 1. Docked compounds are shown in stick format, the interacting residues and FAD cofactor are drawn in line representation. Favorable interactions shown as dashed lines: green—hydrogen bonds, dark pink—π–π stacking interactions, purple—π–sigma interactions, light pink—hydrophobic interactions.

**Figure 8:**
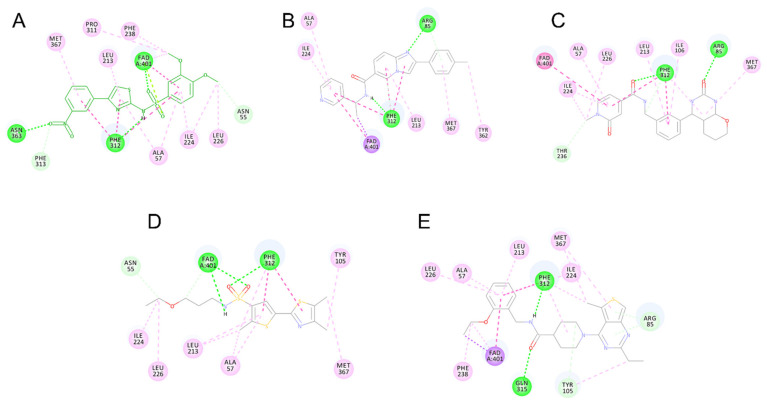
2D interaction pattern of compounds Ro 61-8084 (**A**), VS1 (**B**), VS3 (**C**), VS4 (**D**), VS6 (**E**) in Model 1. Favorable interactions are color coded as follows: green—hydrogen bonds, dark pink—π–π stacking interactions, purple—π–sigma interactions, light pink—hydrophobic interactions. Residues shown in light green form weak van der Waals interactions.

Compounds VS3 and VS4 also share a resemblance with non-substrate effectors (Figure 5C,D), when docked into Model 2, the central placement of the compounds is directed by π–sulfur interactions between aromatic groups and Met367, whereas a pleura of hydrogen and attractive charge interactions with Arg85 and Asn363 occurred via the amide and sulfonamide moiety, respectively. At the same time, VS3 and VS4 were also capable of acquiring a binding mode, similar to that of Ro 61-8084 in Model 1 (Figure 7C,D): the compounds’ aromatic groups were involved in the π–π interactions with Phe312, where the amide or sulfonamide groups form a hydrogen bond with the backbone atoms of Phe312, achieving in general a very similar shape to Ro 61-8084. Additionally, the sulfonamide group of VS4 forms hydrogen bonding with FAD, whereas the VS3 carbonyl forms a hydrogen bond with Arg85 (Figure 8C,D).

The binding mode of compound VS1 closely resembles that of the competitive inhibitor Ro 61-8084 (Figure 7A,B). The compound’s amide bond bends to acquire a similar shape and forms the characteristic π–π and backbone hydrogen-bonding interactions with Phe312. VS1 also forms a hydrogen bond with Arg85 through its imidazopyridine core. Compound VS6 partially acquires both the non-substrate effectors and the competitive inhibitors’ binding modes (Figure 5F and Figure 7E).

**Figure 9:**
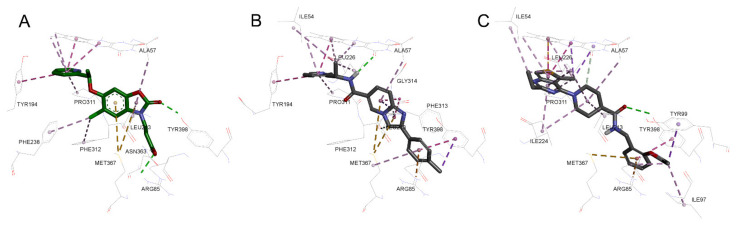
3D docking poses of compounds GSK065 (**A**), VS1 (**B**), VS6 (**C**) in Model 3. Docked compounds are shown in stick format, the interacting residues and FAD cofactor are drawn in line representation. Favorable interactions shown as dashed lines: green—hydrogen bonds, yellow—π–sulfur, orange—π–charge, dark pink—π–π stacking interactions, purple—π–sigma interactions, light pink—hydrophobic interactions.

**Figure 10:**
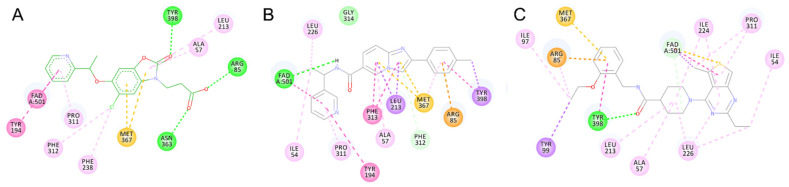
2D interaction patterns of compounds GSK065 (**A**), VS1 (**B**), VS6 (**C**) in Model 3. Favorable interactions are color coded as follows: green—hydrogen bonds, yellow—π–sulfur, orange—π–charge, dark pink—π–π stacking interactions, purple—π–sigma interactions, light pink—hydrophobic interactions. Residues shown in light green form weak van der Waals interactions.

Compounds VS1 and VS6 were the only hit compounds, which were successfully docked into Model 3 and exhibited the characteristic π–π stacking interaction with the tilted FAD moiety (Figure 9). The two compounds also shared π–π stacking interactions with Tyr398 and π–charge interactions with Arg85 (Figure 10).

## 3. Discussion

KMO is an outer mitochondrial membrane-bound protein, which is expressed in peripheral tissues (liver, kidney), in phagocytes (macrophages, monocytes), and in the CNS, predominantly in microglia [42]. Due to the enzyme’s pivotal position in the kynurenine pathway, it is considered as a promising target for regulating most inflammatory neurological diseases by affecting the ratio between excitotoxic and neuroprotective kynurenine pathway metabolites.

It is well-known that the membrane-bound KMO C-terminal transmembrane domain is inherent for both the membrane anchoring and hKMO enzymatic activity [43,44,45], which poses a challenge for the solubilization, purification, and crystallization of the protein in a stable and correctly folded state [46,47]. Nevertheless, the structural basis of KMO inhibition and its enzymatic mechanism have been extensively studied using Sc and PfKMO [33,45,48,49], which exhibit a high rate of sequence identity within the active site, however, overall share very low sequence similarity. PfKMO is considered not to be membrane-bound, whereas ScKMO does not require its C-terminal region for activity.

Since numerous KMO crystal structures have been published, attempts have been made to design KMO inhibitors using computational approaches, especially to find BBB permeable inhibitors with the same promising neuroprotective effects as Ro 61-8084. In the current work, we attempted to exploit Sc and PfKMO crystal structures as templates for homology modeling and pharmacophore-based screening, taking into account the recent advances in elucidating KMO inhibitor binding modes that overcome the damaging production of hydrogen peroxide, which comes from a KMO inhibition side reaction.

The homology models were prepared to account for all of the known KMO inhibitor binding modes. The protein structural validation confirmed the models to be of high quality, whereas docking studies performed with known KMO inhibitors established the ability of the homology models to reproduce known inhibitor binding modes and score them in accordance with their enzymatic activity. Based on the docking modes of representative inhibitors Ro 61-8084, GSK428 and GSK775 protein–ligand complex pharmacophores were generated. The pharmacophore models demonstrated an excellent ability to distinguish true active compounds from decoy molecules in a 50-to-1 enrichment test.

Our virtual screening approach focused on a self-arranged composite compound library, which included readily available for purchase compounds and diverse or CNS-targeting datasets. The workflow was based on primary pharmacophore-based screening, assessment of potential compound toxicity and, in the final step, substantial, detailed docking studies, where compounds were prioritized based on their docking score and resemblance of the binding pattern to that of known active KMO inhibitors.

Out of 95 purchased compounds for screening, only six showed less than 10 µM activity in the fluorescence-based assay. The low hit rate of our screening can be explained by the well-known disadvantage of rigid docking, which cannot account for protein flexibility. In the current work, we did not perform post-docking molecular dynamics simulations, which could improve the quality of decision-making in structure-based approaches [50]. However, we think that a more crucial shortcoming of our homology models and the protein–ligand complex-based pharmacophores is that the initial temple structures do not account for the hKMO C-terminal region. New evidence from the full-length mammalian KMO *in meso* crystal structure suggests that there are drastic differences between Pf and *Rattus norvegicus* KMO C-terminal regions [31]. In contrast to predictions, the Rat KMO C-terminal region contained only one transmembrane helix, whereas the second helix laid laterally along the membrane surface. Furthermore, a dimeric interface inherent for enzyme activity has been reported, which puts into question the appropriateness of modeling KMO as a monomeric unit.

Nevertheless, our virtual screening successfully identified novel KMO inhibitors. Compared to the representative inhibitors, only compound VS1 showed a relatively high 2D similarity to Ro 61-8084 (Table 5), whereas the structural similarity of other hit compounds was 0.6 or lower when checked against all three representative compounds Ro 61-8084, GSK428, and GSK775. Analysis of the compounds’ structure and binding modes suggests that compounds VS1 and VS6 could bind to KMO as competitive inhibitors via both of the known mechanisms (apo structure binding or FAD-tilting). Compounds VS2 and VS5 exhibit a typical non-substrate effector binding pattern, making them prone to hydrogen peroxide production. However, these compounds can be considered the core of an inhibitor series, which, if modified by adding aromatic groups such as alkoxyl-linked pyridyl could become competitive inhibitors with altered binding kinetics [24,35].

The Discovery Studio ADMET-BBB model predicted compounds VS1 and VS6 to be potentially BBB permeable, therefore, they were our prioritized hit compounds for further chemical modification to improve the compounds’ inhibition activity and drug-likeness profile.

## 4. Materials and Methods

### 4.1. hKMO Homology Modeling

The target amino acid sequence of hKMO for homology modeling was retrieved from the UniProtKB database entry O15229 (https://www.uniprot.org/). The following template crystal structures were chosen from the protein data bank (https://www.rcsb.org/) as the best representatives of different KMO inhibitor binding modes: 5x6r (Model 1), 5nak (Model 2), and 5nag (Model 3). When several crystal structures were available to represent the same protein conformation and/or bound ligand, structures with lower resolution were preferred. A detailed description of the models’ representative conformations and bound ligands are provided in the Results section. For further information on the template parameters, please refer to Table S1.

Homology modeling was performed using the Discovery Studio v.18 software package (Accelrys, San Diego, CA, USA) [51] based on the sequence alignment in Figure S1. For all template structures, chain A was chosen to perform homology modeling. In chain B of the 5x6r structure, the C-terminal α-helix shifted outward, making the active site inappropriately solvent-exposed. In 5nak and 5nag structures, chains A and B were identical, so chain A was chosen systematically. Template protein preparation for Models 1 and 2 included deleting alternate residue conformations, removing water molecules, adding missing protein loops, completing all side chains, adding hydrogen atoms, and protonating titratable residues. The added missing loops (total five loops for Model 1 and 1 for Model 2) were further optimized by using the Looper refinement tool [52]. After protein preparation, homology models were created using MODELER [53]. The coordinates of the FAD cofactor and bound ligands were preserved and copied directly to the homology models. 

A total of 10 homology models were prepared for each template. The best model was selected by considering the PDF (probability density function) total energy, heavy atom RMSD (root-mean-square deviation) between the model and template and Profiles-3D score [54] of important protein regions. Homology model loop regions with low 3D profile scores and invalid dihedral angles were optimized the same way as during protein preparation. To fully optimize and relax the final model structure, restrained energy minimization within 0.3 Å heavy atom displacement was performed in Schrodinger Maestro [55]. The structure quality of the final model was assessed based on the Ramachandran plot, total Profiles-3D score, and the Maestro protein reliability report [55], which checks metrics such as the average B-factors, steric clashes, protein packing, and peptide planarity, bond angle and length deviations, improper torsions, etc.

Model 3 was prepared by replacing FAD molecules and the bound ligand in the final Model 2 with those from the 5nag crystal structure. After small molecule replacement, the Arg52-Leu56 loop was optimized and minimized, followed by restrained energy minimization within a 0.3 Å heavy atom displacement of the whole protein structure. This simplified model preparation method was chosen, because according to the literature, upon FAD tilting, the active site residues remain unaltered, and only slight rearrangements occur below the FAD isoalloxazine ring system [35]. The final structure quality was verified the same way as the previous models.

### 4.2. Docking Protocol

All active KMO inhibitors were first drawn in ChemDraw Professional 16.0, and afterward, imported into Discovery Studio, where the ligands were prepared. Ligand preparation included the generation of the ligand 3D conformations, enumerating all isomers (in cases when stereochemistry was not indicated in the publications), tautomers, and appropriate ionization states at pH 7. Full ligand energy minimization was performed under the general-purpose all-atom forcefield CHARMm. Molecular docking was performed using the Discovery Studio CDOCKER docking algorithm [56]. The optimal binding sites for docking were defined as co-crystallized ligand-centered spheres with the following radii: Model 1—9.25 Å, Model 2—8 Å, Model 3—10 Å.

The docking protocol was validated based on docking pose accuracy and scoring ability. The docking pose accuracy corresponded to heavy atom RMSD between the top-scoring dock pose and a corresponding ligand co-crystal pose. A total of 11 KMO inhibitor-bound crystal structures were chosen as verification standards (5x6r, 5nak, 4j36, 5n7t, 5nab, 5mzc, 5mzi, 5mzk, 5nag, 5nah, 5nae). The model scoring ability refers to the correlation between the inhibitor activity and the docking score of its first dock pose. A total of 45 competitive inhibitor analogues (Type II) from three different scaffolds were chosen to validate Model 1, 30 non-substrate effectors (Type I) from two different scaffolds were chosen to validate Model 2, and 14 competitive inhibitor analogues (Type II) from one scaffold were chosen to validate Model 3. The compounds were chosen based on several factors: their 2D structural similarity to the co-crystallized inhibitors, available experimental confirmation of hydrogen peroxide production, and overall set activity range coverage. All of the active compounds, which were included in the docking score validation sets, are highlighted in bold in Table S3.

### 4.3. Pharmacophore Generation and Validation

The Discovery Studio Interaction Pharmacophore Generation Tool was used to create and validate pharmacophores from the non-bond protein–ligand interactions between each homology model and a chosen representative inhibitor docking pose. KMO inhibitors Ro 61-8084 [18], GSK428, and GSK775 [35] were selected as representative compounds for Models 1–3, respectively. The choice of compounds was based on similarity to the crystal structure pose, inhibitor activity and –CDOCKER docking score. During pharmacophore generation, hydrogen bond donor, hydrogen bond acceptor, hydrophobic, ring aromatic, negative and positive ionizable ligand feature types were included and cross-checked for correspondence to the binding pocket features. The matched features were afterward mixed and selected to create all possible four to six feature options. The top 10 pharmacophores for each protein–ligand complex were outputted based on the highest selectivity score predicted by the software’s embedded genetic function approximation (GFA) algorithm. The latter is a crude estimation of the uniqueness of the feature combination compared to a standard built-in library of pharmacophore models.

All top 10 pharmacophores for each model were validated based on the scoring difference between active and non-active compounds. The validation set of true active compounds was the same as during the docking protocol validation, but with an increased number of compounds (refer to Table S3). In the absence of a published large set of true inactive compounds, decoy molecules were generated from the active compound validation set using the DUD-E online service (http://dude.docking.org/). Decoy molecules are compounds that have a similar molecular weight, logP, number of rotatable bonds, and other features to the input actives but low 2D structural similarity [57]. For the current study, we generated a validation dataset in which there were at least 50 decoy molecules per active compound. Since Model 3 is represented by a scarce number of active compounds, the number of decoys was increased to 100 per active compound. It should be noted that for Model 1, 15% of decoys failed the 2D structure generation from the SMILES format and ligand preparation steps, whereas for Models 2 and 3, the loss was insignificant (less than 5%).

The pharmacophores were evaluated based on their sensitivity and specificity.

(1) $$\mathrm{Sensitivity}=\mathrm{TP}/\left(\mathrm{TP}+\mathrm{FP}\right)$$

(2) $$\mathrm{Specificity}=\mathrm{TN}/\left(\mathrm{TN}+\mathrm{FN}\right)$$

where TP is the true positives (actives that were correctly predicted active), TN is the true negatives (decoys that were correctly predicted inactive), FP is the false positives (decoys that were incorrectly predicted active), and FN is the false negatives (actives that were incorrectly predicted inactive).

The pharmacophore validation also included plotting of the receiver operating characteristic (ROC) curves. The area under the curve (AUC) of the ROC accounts for both the sensitivity and specificity of the pharmacophore in a single characteristic.

### 4.4. Compound Library Generation

For the purpose of the current study, we aimed to screen against a diverse set of readily available, potentially CNS-penetrant compounds. A total of 12 compound libraries from four different vendors were accessed in May 2019, downloaded, and merged into a single composite compound library, according to Table 6. All compounds were included in a 3D conformational database created in Discovery Studio in which for each structure 255 3D conformations were generated.

### 4.5. Virtual Screening

The 3D pharmacophore search of the complete compound database was performed separately for each of the three prepared and validated pharmacophore models, as summarized in Figure 4. In the first screening step, we applied Discovery Studio’s Search DB protocol, which was optimized for speedy and efficient single pharmacophore screening. In the second step, libdock was applied to all compounds of the search results to eliminate those compounds that do not fit the ligand binding pocket despite compliance with the pharmacophore features. As a third step, potentially toxic compounds were eliminated based on Discovery Studio extensible toxicity prediction QSAR models. Compounds with a predicted toxicity probability of more than 0.65 were eliminated. The prediction algorithm considered carcinogenic potency TD50, Ames mutagenicity, and rat oral LD_50_ properties. Next, the previously validated CDOCKER docking algorithm was applied to the predicted non-toxic compounds. In the case of pharmacophore Models 2 and 3, all compounds (654 and 672, respectively) were docked, whereas for pharmacophore model 1 out of 60,850 compounds, 6925 were selected based on the preliminary docking results: these compounds had a fit value of more than 2.5 and exhibited at least three hydrophobic interactions with residue Phe312 and at least one favorable interaction with Arg85. The final compound selection was made based on visual inspection of the dock pose interactions among the top-scoring 25% of the compounds. For Model 1 screening results, favorable interactions with residues Arg85, Phe312, Asn363, and Met367 were considered important. For Models 2 and 3, the selection criteria included favorable interactions with Arg85, Tyr99, Tyr398, Ala57, or π–π stacking interactions with FAD, respectively. The final selection compounds were grouped based on their functional-class fingerprints (FCFP_6), and the best representative compounds from each group were purchased for biological activity testing.

### 4.6. Structure Similarity and BBB Permeability Calculations

The novelty of the discovered hit compound molecules was assessed by calculating the 2D Tanimoto similarity coefficient between the hit compounds and the representative inhibitors used to create the pharmacophore screening models: Ro 61-8084, GSK428, and GSK775. All hits were checked against all representative inhibitors regardless of their ability to acquire a similar binding mode or not in the docking studies. The highest Tanimoto coefficient served as the novelty assessment criteria. 

Hit compound BBB permeability was estimated in the Discovery Studio software package using the Calculate ADMET Descriptors tool. The prediction model was previously validated on a dataset of 881 CNS compounds from the CMC dataset [58]. The prediction model was based on quantitative linear regression between BBB permeability and the compounds 2D polar surface area (PSA) and logP. Compounds with calculate log B more than 0 are considered high BBB penetrants, capable of having a blood–brain ratio between 1:1 and 5:1. If the logBB is less than 0.52, then BBB permeability is considered low, with a blood–brain ratio less than 0.3:1.

### 4.7. KMO Inhibitor Screening Assay 

The BPS Bioscience KMO Screening Kit (79513-2) was used to measure the hKMO enzyme inhibition. The assay is based on the consumption of the fluorescent enzymatic cofactor NADPH during the KMO catalytical reaction [41]. Self-fluorescent inhibitor compounds were first excluded from the screening if their 10 µM solution exhibited a substantial fluorescence signal compared to the blank. The screening was performed in a 96-well plate, with all samples and controls duplicated. A total of 50 µL of purified KMO protein with C-terminal His-FLAG-tag was mixed in 40 µL of substrate mixture (containing NADPH, L-kyn, and the assay buffer) and 10 µL of inhibitor solutions. The time-dependent NADPH absorbance change was measured at λ = 340 nm and compared with the blank negative control after incubation at room temperature. Ro 61-8084 served as a positive control to confirm the correct setup of the screening assay. Compounds that showed IC_50_ less than 10 µM were considered inactive.

## Figures and Tables

**Table 1:** Final structure quality validation of the hKMO homology models.

Validation Parameter	Model 1	Model 2	Model 3
Ramachandran plot residues in allowed region, %	95.5	97	96.7
PDF total energy	−8089.32	2595	
RMSD to template, Å	0.939	0.503	
3D-Profile score, verify score/expected high score	165.55/170.026	169.78/202.672	171.19/202.672

**Table 2:** Protein–ligand complex-based pharmacophores and their validation.

Pharmacophore Characteristic	Model 1	Model 2	Model 3
	Pharmacophore Ro 61-8084 Based	Pharmacophore GSK428 Based	Pharmacophore GSK775 Based
Total Features	5	5	5
Feature Set *	AAADH	AAHNS	AHHNS
Sensitivity	0.846	0.956	1.00
Specificity	0.816	0.843	0.981
AUC **	0.858	0.951	0.999

* A—hydrogen bond acceptor feature, D—hydrogen bond donor feature, H—hydrophobic feature, N—negative ionizable feature, S—shape feature. ** AUC—area under the receiver operating characteristic (ROC) plot.

**Table 3:** Number of compounds that passed the screening criteria at each step.

№	Screening Step	Model 1	Model 2	Model3	Total
1	Pharmacophore filter	143,205	1043	1109	145,357
2	Libdock filter	120,006	1039	1058	122,103
3	Toxicity filter	61,067	654	672	62,393
4	Selected for docking	6925	654	672	8251
5	Final Selection	163	50	42	255

**Table 4:** Structures and activity of identified novel KMO inhibitors and reference compounds.

Vendor Code	Vendor	Structure	MW	% Inhibition, µM			-CDOCKER Score, kcal/mol		
				10	1	0.1	Model 1	Model 2	Model 3
P323-0389	Chemdiv	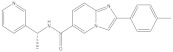 VS1	356.43	97.8	21.3	6.3	27.49	28.01	27.56
D715-2857	Chemdiv	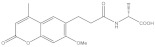 VS2	332.33	109.4	23.9	3.1	* ND	40.48	ND
Z354-0210	Chemdiv	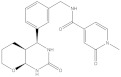 VS3	396.45	107.5	15.1	3.6	37.99	37.53	ND
L921-0479	Chemdiv	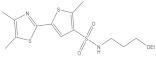 VS4	374.53	109.7	21.2	8.4	42.11	42.32	ND
BDE 33672567	Asinex	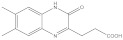 VS5	246.27	66.3	17.8	16.8	ND	45.25	ND
F6548-0495	Life Chemical	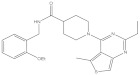 VS6	438.59	60.4	27.1	4.3	41.52	25.82	35.53
		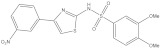 Ro 61-8084	421.449	97.3	60.5	7.5	23.43	ND	ND
		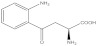 L-kyn	208.22				ND	46.09	ND
		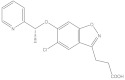 GSK065	376.77				ND	ND	43.07

* ND—docking score not determined because the compound binding mode did not resemble the shape and interaction pattern of the model inhibitor.

**Table 5:** Assessment of the hit compounds’ inhibition type, BBB permeability, and structural novelty.

Comp	Presumed Binding Mode	Tanimoto Similarity	logBB	Penetration Ability
VS1	Competitive inhibitor	0.709	−0.057	Medium
VS2	Non-substrate effector	0.532	−1.574	Low
VS3	Undetermined, both possible	0.395	−1.677	Low
VS4	Undetermined, both possible	0.482	−0.641	Low
VS5	Non-substrate effector	0.605	−1.42	Low
VS6	Competitive inhibitor	0.596	0.02	High

**Table 6:** List of compound libraries included in the virtual screening.

Vendor	Library Name	Total Compounds	Available at
ChemDiv	CNS Library	22,306	https://www.chemdiv.com/Accessed Accessed 17 May 2019
	Representative Diversity Library	150,000	
	3D-Biodiversity Library	30,073	
	New Chemistry (NC) Library	325,760	
	Smart Library	52,504	
Life Chemical	Stock HTS compounds	45,589	https://lifechemicals.com/Accessed Accessed 17 May 2019
	CNS screening Library	7024	
	Low MW Fragment Library	36,080	
Asinex	Signature Library	7815	http://www.asinex.com/Accessed Accessed 6 May 2019
	BioDesign Library	195,039	
Specs	Screening Compounds (10mg)	210,419	https://www.specs.net/Accessed Accessed 7 May 2019
	Building blocks Library	8985	
	Total	1,091,594	

## Data Availability

All data are available from the author.

## Supplementary Materials

The following are available online. Figure S1: Alignment of KMO sequences for human, Sc, and Pf, Figure S2: Protein structure validation for homology Models 1–3, Figure S3: Validation of homology models via docking of known active inhibitors, Figure S4: ROC plots for Ro 61-8084, GSK428, and GSK775 based pharmacophores, Figure S5: Obtained dose-response curve for Ro 61-8084 in the KMO fluorescence assay, Table S1: Homology modeling template parameters, Table S2: Homology model docking accuracy, Table S3: Compound validation sets for the docking protocol and protein–ligand complex pharmacophore performance, Table S4: Results of the KMO screening assay (excluding self-fluorescent compounds).
