# Supplementary material for: Pharmacophore-Based Virtual Screening of Novel Competitive Inhibitors of the Neurodegenerative Disease Target Kynurenine-3-Monooxygenase
Source: Molecules. 2021 May 31;26(11):3314. doi: 10.3390/molecules26113314 (PMC8199213; doi:10.3390/molecules26113314)
Supplement: Supplementary file 1 [file molecules-26-03314-s001.zip › molecules-1211113-supplementary.pdf]

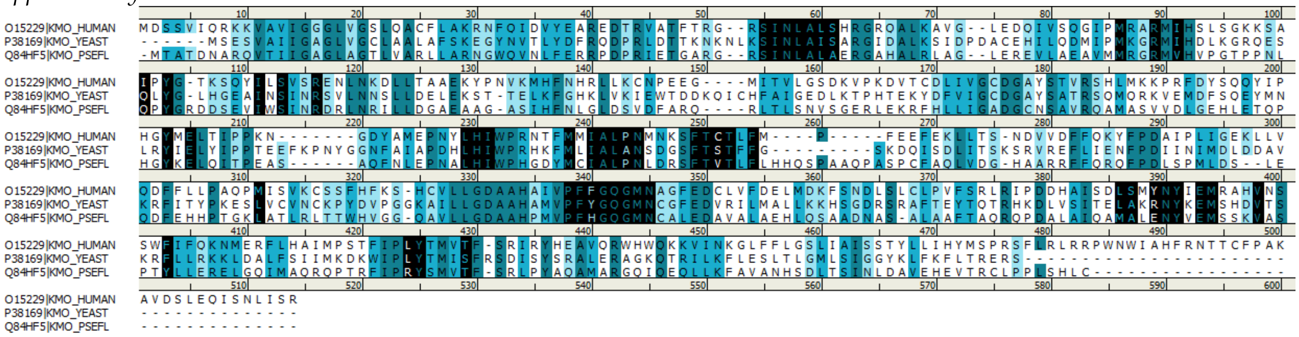

Figure S1. Alignment of KMO sequences for Human, Scacharmyces cerevisiae and Pseudomonas fluorescens. Amino acids colored according to their similarity. Residues within 6 Å of the ligand biding site are highlighted in black.

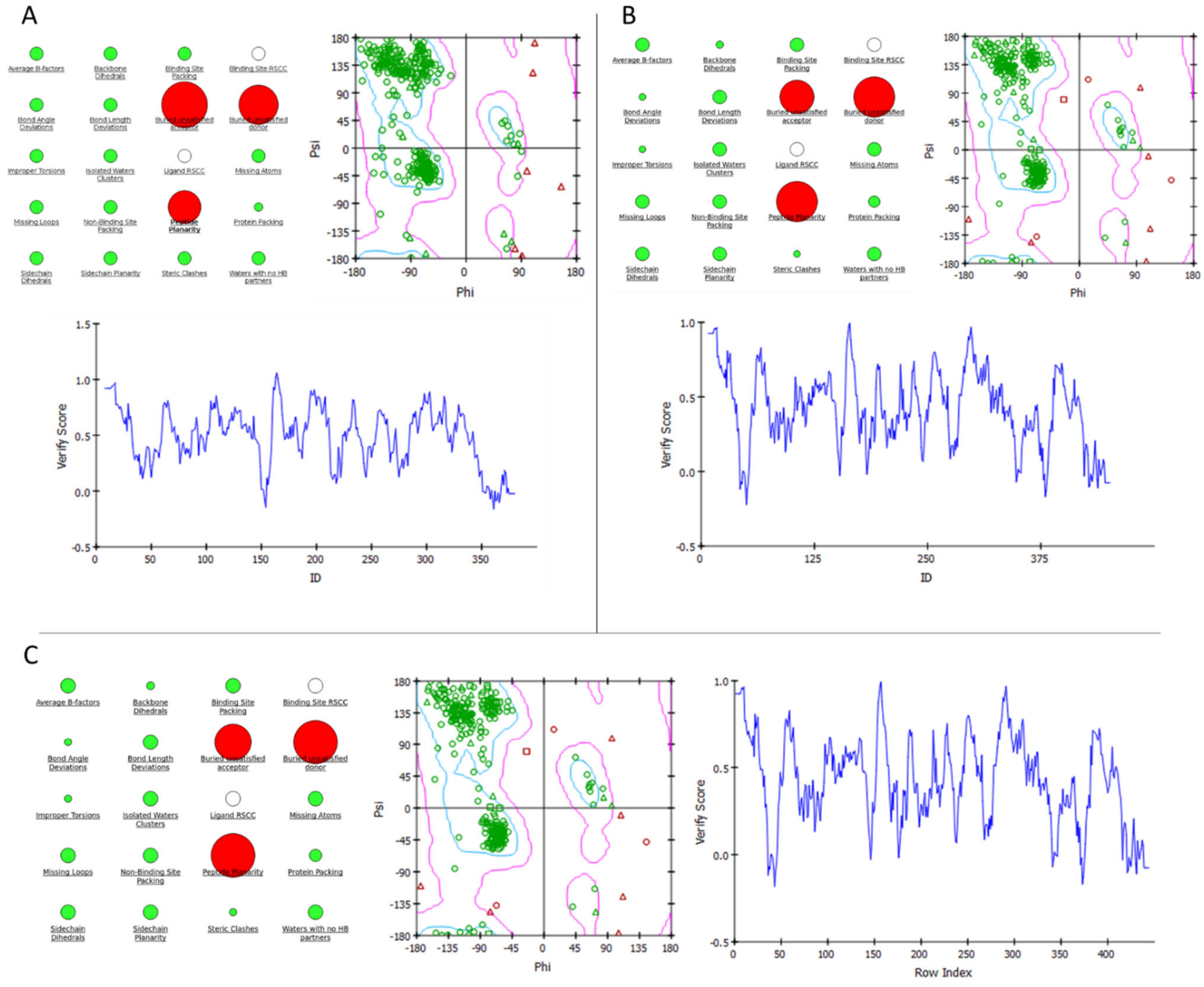

Figure S2. Maestro Protein Reliability Report, Ramachandran Plot and 3D Profiles score line plot for Homology Models 1-3 (A-C respectively).

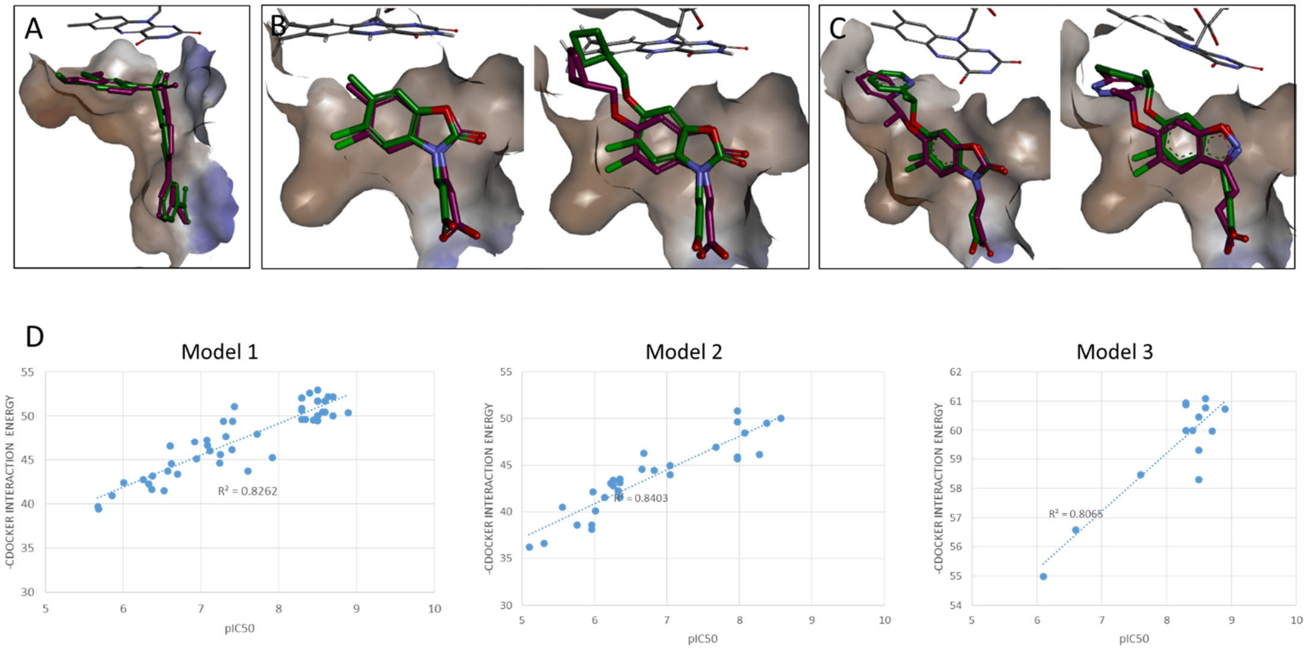

Figure 3. Validation of Homology Models via docking of known active inhibitors. (a) Overlay of docking and crystal structure pose of Ro 61-8084 in Model 1; (b) Overlay of docking and crystal structure pose of GSK428 (accurate) and 5MZK (least accurate) in Model 2; (c) Overlay of docking and crystal structure pose of GSK775 (most accurate) and GSK366 (least accurate) in Model 3. Crystal binding poses represented as green sticks, docking poses – magenta sticks, FAD shown in grey stick format. (d) Correlation plots between KMO inhibitor activity and docking score.

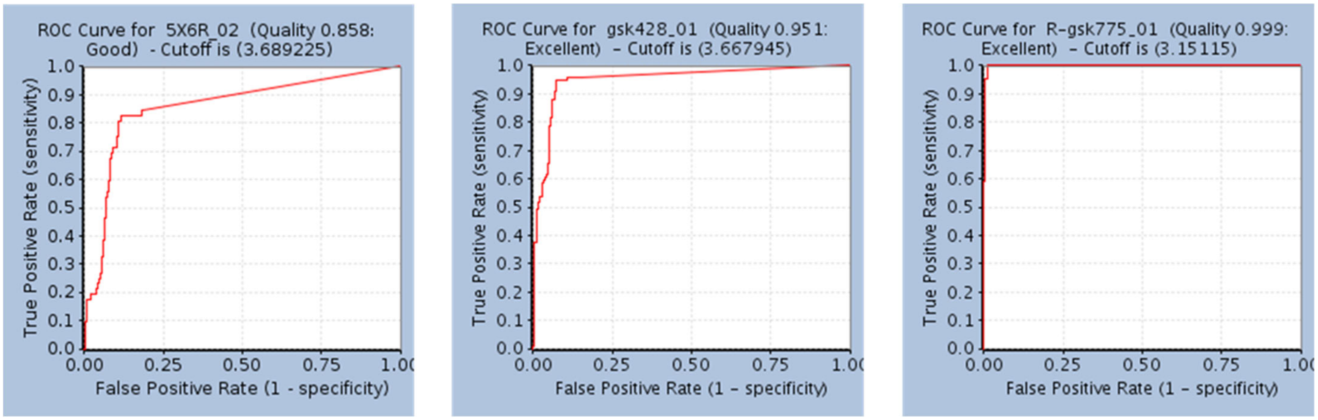

Figure S4. Receiver Operating Characteristic (ROC) plots for (a) Ro 61-8084 based pharmacophore (Model 1); (b) GSK428 based pharmacophore (Model 2); (c) GSK775 based pharmacophore (Model 3).

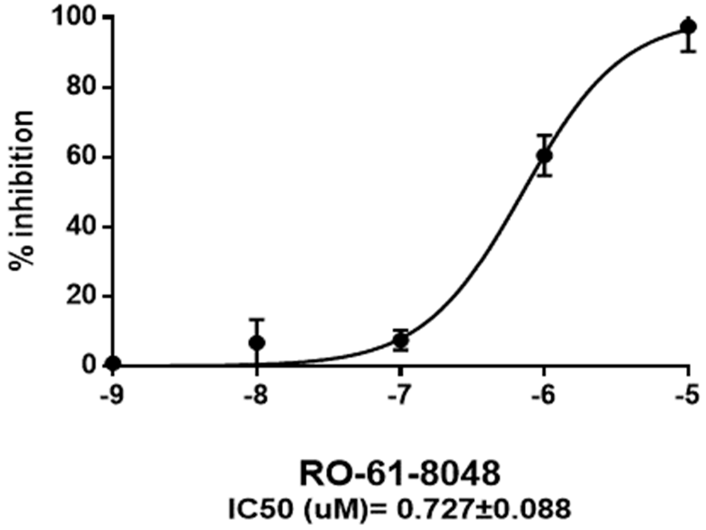

Figure S5. Obtained dose–response curve for Ro-61-8048 in the KMO fluorescence assay.

Table S1. Homology Modelling Template Parameters.

		**Model 1**	Model 2	Model 3	
**Model Purpose**		**Competitive Inhibitor Model**	Non-substrate Effector Model	Competitive Inhibitor Model	
Template Info	PDB code	5X6R	5NAK	5NAK	5NAG
	Organism	*Saccharomyces cerevisiae*	*Pseudomonas fluorescens*	*Pseudomonas fluorescens*	
	Method	X-RAY DIFFRACTION	X-RAY DIFFRACTION	X-RAY DIFFRACTION	
	Resolution	1.911 Å	1.5 Å	1.5 Å	1.68 Å
	Ligands	FAD, Ro 61-8048	FAD, L-kyn	FAD, L-kyn	FAD, GSK065
	Residues	Met1-Lys390	Ala7-Leu457	Ala7-Leu457	-
Comment		Conformation of loop 321-PFYGQ-325 corresponds to *Sc*KMO apo structure, C-terminal alpha helix is not displaced	Conformation of loop 318-PFHGQ-322 and C-terminal region corresponds to L-kyn bound state.	FAD in Model 2 replaced with "tilted" FAD from 5NAG.	

Table S2. Homology Model Docking Accuracy.

**Model 1**			Model 2			Model 3		
**Competitive Inhibitor Model**			Non-substrate Effector Model			Competitive Inhibitor Model		
Co-crystal ligand	Reference structure PDB code	Dock pose vs Crystal pose RMSD, Å	Co-crystal ligand	Reference structure PDB code	Dock pose vs Crystal pose RMSD, Å	Co-crystal ligand	Reference structure PDB code	Dock pose vs Crystal pose RMSD, Å
Ro 61-8048	5X6R	0.5739	L-Kyn	5NAK	0.4324	GSK065	5NAG	0.8381
			UPF648	4J36	0.5892			
			GSK180	5N7T	0.5792	GSK366	5NAH	1.4051
			GSK428	5NAB	0.6043			
			GSK-OEt	5MZC	0.5832	GSK775	5NAE	0.8034
			GSK-OcPr	5MZI	0.9371			
			GSK-OCcBu	5MZK	0.9768			

Table S3. Compound Validation sets for the Docking Protocol and Protein-Ligand Complex Pharmacophore Performance*.

Model 1

№	Comp name	Smiles	Molecular Formula	KMO pIC50	KMO IC50, nM	reference
1	2	[O-]C(=O)c1cc(on1)c2cccc(c2)[N+](=O)[O-]	C10 H5 N2 O5	5.6	2680	[1]
2	3	[O-][N+](=O)c1cccc(c1)c2onc(c2)c3nnn[nH]3	C10 H6 N6 O3	5.9	1160	
3	5	Cc1ccc(cc1)S(=O)(=O)Nc2nc(cs2)c3ccc(O)c(C)c3	C17 H16 N2 O3 S2	6.9	114	
4	6	Cc1ccc(cc1)S(=O)(=O)Nc2nc(cs2)c3ccccc3	C16 H14 N2 O2 S2	6.3	470	
5	7	COc1ccc(cc1)c2csc(NS(=O)(=O)c3ccc(C)cc3)n2	C17 H16 N2 O3 S2	6.6	240	
6	8	Cc1ccc(cc1)S(=O)(=O)Nc2nc(cs2)c3ccc(Cl)cc3	C16 H13 Cl N2 O2 S2	7.3	56	
7	9	Cc1ccc(cc1)c2csc(NS(=O)(=O)c3ccc(C)cc3)n2	C17 H16 N2 O2 S2	7.1	76	
8	11	Cc1ccc(cc1)S(=O)(=O)Nc2nc(cs2)c3cccc(c3)[N+](=O)[O-]	C16 H13 N3 O4 S2	7.3	48	
9	12	[O-][N+](=O)c1cccc(c1)c2csc(NS(=O)(=O)c3ccccc3)n2	C15 H11 N3 O4 S2	6.7	200	
10	13	[O-][N+](=O)c1cccc(c1)c2csc(NS(=O)(=O)c3ccc(Cl)c(Cl)c3)n2	C15 H9 Cl2 N3 O4 S2	6.9	120	
11	14	[O-][N+](=O)c1cccc(c1)c2csc(NS(=O)(=O)c3ccc(Cl)cc3)n2	C15 H10 Cl N3 O4 S2	7.1	84	
12	15	COc1ccc(cc1)S(=O)(=O)Nc2nc(cs2)c3cccc(c3)[N+](=O)[O-]	C16 H13 N3 O5 S2	7.3	51	
13	17	Nc1ccc(cc1)S(=O)(=O)Nc2nc(cs2)c3cccc(c3)[N+](=O)[O-]	C15 H12 N4 O4 S2	7.4	40	
14	18	CC(C)c1ccc(cc1)S(=O)(=O)Nc2nc(cs2)c3cccc(c3)[N+](=O)[O-]	C18 H17 N3 O4 S2	6.0	990	
15	19	COc1ccc(cc1OC)S(=O)(=O)Nc2nc(cs2)c3cc(ccc3F)C(F)(F)F	C18 H14 F4 N2 O4 S2	7.4	39	
16	20	Nc1ccc(cc1)S(=O)(=O)Nc2nc(cs2)c3cc(ccc3F)C(F)(F)F	C16 H11 F4 N3 O2 S2	7.7	19	
17	11	[O-]C(=O)CCN1C(=O)Oc2cc(OCCN3CCCC3)c(Cl)cc12	C16 H18 Cl N2 O5	6.1	794	[2]
18	12	[O-]C(=O)CCN1C(=O)Oc2cc(OC(=O)c3ccccc3)c(Cl)cc12	C17 H11 Cl N O6	7.6	25.1	
19	13	[O-]C(=O)CCN1C(=O)Oc2cc(OCc3ccccn3)c(Cl)cc12	C16 H12 Cl N2 O5	8.5	3.16	
20	15	C[C@@H](Oc1cc2OC(=O)N(CCC(=O)[O-])c2cc1Cl)c3ccccn3	C17 H14 Cl N2 O5	8.6	2.51	
21	16	C[C@H](Oc1cc2OC(=O)N(CCC(=O)[O-])c2cc1Cl)c3ccccn3	C17 H14 Cl N2 O5	6.6	251	
22	17	C[C@@H](Oc1cc2OC(=O)N(CCC(=O)[O-])c2cc1Cl)c3occn3	C15 H12 Cl N2 O6	8.5	3.16	
23	18	C[C@@H](Oc1cc2OC(=O)N(CCC(=O)[O-])c2cc1Cl)c3ncccn3	C16 H13 Cl N3 O5	8.3	5.01	
24	19	C[C@@H](Oc1cc2OC(=O)N(CCC(=O)[O-])c2cc1Cl)c3cccnn3	C16 H13 Cl N3 O5	8.9	1.26	
25	20	C[C@@H](Oc1cc2OC(=O)N(CCC(=O)[O-])c2cc1Cl)c3cc(C)ccn3	C18 H16 Cl N2 O5	8.4	3.98	
26	21	C[C@@H](Oc1cc2OC(=O)N(CCC(=O)[O-])c2cc1Cl)c3ccc(C)cn3	C18 H16 Cl N2 O5	8.5	3.16	
27	22	C[C@@H](Oc1cc2OC(=O)N(CCC(=O)[O-])c2cc1Cl)c3ccc(Cl)cn3	C17 H13 Cl2 N2 O5	8.5	3.16	
28	23	C[C@@H](Oc1cc2OC(=O)N(CCC(=O)[O-])c2cc1Cl)c3ccc(F)cn3	C17 H13 Cl F N2 O5	8.6	2.51	
29	24	C[C@@H](Oc1cc2OC(=O)N(CCC(=O)[O-])c2cc1Cl)c3cccc(C)n3	C18 H16 Cl N2 O5	8.3	5.01	
30	25	C[C@@H](Oc1cc2OCC(=O)N(CCC(=O)[O-])c2cc1Cl)c3ccccn3	C18 H16 Cl N2 O5	8.3	5.01	
31	26	C[C@@H](Oc1cc2ccn(CCC(=O)[O-])c2cc1Cl)c3ccccn3	C18 H16 Cl N2 O3	8.3	5.01	
32	27	C[C@@H](Oc1cc2cnn(CCC(=O)[O-])c2cc1Cl)c3ccccn3	C17 H15 Cl N3 O3	8.5	3.16	
33	28	C[C@@H](Oc1cc2onc(CCC(=O)[O-])c2cc1Cl)c3ccccn3	C17 H14 Cl N2 O4	8.3	5.01	
34	29	C[C@@H](Oc1cc2SC(=O)N(CCC(=O)[O-])c2cc1Cl)c3ccccn3	C17 H14 Cl N2 O4 S	8.7	2.00	
35	30	C[C@@H](Oc1cc2onc(CCC(=O)[O-])c2cc1Cl)c3ccc(C)cn3	C18 H16 Cl N2 O4	8.5	3.16	
36	31	C[C@@H](Oc1cc2onc(CCC(=O)[O-])c2cc1Cl)c3ccc(Cl)cn3	C17 H13 Cl2 N2 O4	8.5	3.16	
37	32	C[C@@H](Oc1cc2onc(CCC(=O)[O-])c2cc1Cl)c3ccc(F)cn3	C17 H13 Cl F N2 O4	8.5	3.16	

**38**	**33**	**C[C@@H](Oc1cc2onc(CCC(=O)[O-])c2cc1Cl)c3ccc(C)nn3**	**C17 H15 Cl N3 O4**	**8.7**	**2.00**	
**39**	**38**	**Clc1ccc(cc1Cl)c2cc(NS(=O)(=O)c3ccccc3)ncn2**	**C16 H11 Cl2 N3 O2 S**	**6.6**	**270**	
**40**	**48**	**[O-]C(=O)c1cc(ncn1)c2cccc(c2)c3ccccc3**	**C17 H11 N2 O2**	**5.7**	**2130**	
**41**	**51**	**Clc1ccc(cc1Cl)c2cc(ncn2)C(=O)NS(=O)(=O)c3ccccc3**	**C17 H11 Cl2 N3 O3 S**	**7.6**	**25**	[3]
**42**	**70**	**[O-]C(=O)c1cc(ncn1)c2ccc(OCCN3CCOCC3)c(Cl)c2**	**C17 H17 Cl N3 O4**	**7.1**	**83**	
**43**	**78**	**[O-]C(=O)c1cc(ncn1)c2ccc(OC3CCC3)c(Cl)c2**	**C15 H12 Cl N2 O3**	**7.9**	**12**	
**44**	**80**	**[O-]C(=O)c1cc(ncn1)c2ccc(OC3CCCC3)c(Cl)c2**	**C16 H14 Cl N2 O3**	**7.2**	**57**	
**45**	**81**	**[O-]C(=O)c1cc(ncn1)c2ccc(OC3CCCCC3)c(Cl)c2**	**C17 H16 Cl N2 O3**	**6.4**	**430**	
№	Comp name	Smiles	Molecular Formula	% Inhibi-tion	KMO Ki, µM	reference
46	GM141	COc1ccc(cc1OC)C(=O)Nc2onc(n2)c3cccc(c3)[N+](=O)[O-]	C17 H14 N4 O6	37.4	1.300	
47	GM195	COc1ccc(C(=O)Nc2onc(n2)c3cccc(c3)[N+](=O)[O-])cc1OC	C18 H16 N4 O6	32.0	1.820	
48	GM303	Clc1ccc(cc1Cl)c2noc(NC(=O)c3ccccc3)n2	C15 H9 Cl2 N3 O2	95.0	0.115	
49	GM308	Brc1cccc(c1)c2noc(NC(=O)c3ccccc3)n2	C15 H10 Br N3 O2	13.0	1.581	
50	GM340	Clc1cccc(c1)c2noc(NC(=O)c3ccccc3)n2	C15 H10 Cl N3 O2	22.0	2.770	[4]
51	GM347	COc1ccc(cc1OC)C(=O)Nc2onc(n2)c3ccc(Cl)c(Cl)c3	C17 H13 Cl2 N3 O4	94.4	0.065	
52	GM466	COc1ccc(cc1OC)C(=O)Nc2onc(n2)c3cccc(Cl)c3	C17 H14 Cl N3 O4	56.4	0.782	
53	GM760	CCOP(=O)(OCC)Oc1cc(ccc1OC)C(=O)Nc2onc(n2)c3ccc(Cl)c(Cl)c3	C20 H20 Cl2 N3 O7 P	91.6	0.020	

Model 2

№	Comp name	Smiles	Molecular Formula	KMO pIC50	KMO IC50, nM	reference
**1**	**1**	**[NH3+][C@@H](CC(=O)c1ccc(Cl)c(Cl)c1)C(=O)[O-]**	**C10 H9 Cl2 N O3**	**6.5**	**330**	
**2**	**2**	**[O-]C(=O)CCC(=O)c1ccc(Cl)c(Cl)c1**	**C10 H7 Cl2 O3**	**5.4**	**3900**	
**3**	**4**	**C[C@@H](CC(=O)c1ccc(Cl)c(Cl)c1)C(=O)[O-]**	**C11 H9 Cl2 O3**	**5.2**	**6900**	
**4**	**5**	**CO[C@@H](CC(=O)c1ccc(Cl)c(Cl)c1)C(=O)[O-]**	**C11 H9 Cl2 O4**	**5.2**	**6900**	
**5**	**6**	**[O-]C(=O)\C=C\C(=O)c1ccc(Cl)c(Cl)c1**	**C10 H5 Cl2 O3**	**4.4**	**36000**	
**6**	**7**	**[O-]C(=O)[C@@H](Cl)CC(=O)c1ccc(Cl)c(Cl)c1**	**C10 H6 Cl3 O3**	**4.8**	**14800**	
**7**	**9**	**[O-]C(=O)[C@@H](CC(=O)c1ccc(Cl)c(Cl)c1)c2ccccc2**	**C10 H7 Cl2 O4**	**4.7**	**19500**	
**8**	**15**	**[O-]C(=O)\C=C\C(=O)c1ccc(Cl)c(Cl)c1**	**C16 H11 Cl2 O3**	**5.0**	**10400**	
**9**	**16**	**[O-]C(=O)\C=C\C(=O)c1ccc(F)c(F)c1**	**C17 H13 Cl2 O3**	**4.7**	**21100**	
**10**	**17**	**C\C(=C/C(=O)c1ccc(Cl)c(Cl)c1)\C(=O)[O-]**	**C10 H5 F2 O3**	**4.9**	**11300**	
**11**	**18**	**O\C(=C\C(=O)c1ccc(Cl)c(Cl)c1)\C(=O)[O-]**	**C11 H7 Cl2 O3**	**5.2**	**6400**	[5]
**12**	**19**	**O\C(=C\C(=O)c1ccc(F)c(F)c1)\C(=O)[O-]**	**C10 H5 Cl2 O4**	**5.9**	**1200**	
**13**	**20**	**O[C@@H](CC(=O)c1cccc(Cl)c1)C(=O)[O-]**	**C10 H5 F2 O4**	**6.0**	**1100**	
**14**	**21**	**O[C@@H](CC(=O)c1cccc(F)c1)C(=O)[O-]**	**C10 H8 Cl O4**	**5.0**	**9100**	
**15**	**22**	**O[C@@H](CC(=O)c1cccc(c1)[N+](=O)[O-])C(=O)[O-]**	**C10 H8 F O4**	**4.9**	**11500**	
**16**	**23**	**O[C@@H](CC(=O)c1ccc(F)c(F)c1)C(=O)[O-]**	**C10 H8 N O6**	**5.5**	**3000**	
**17**	**25**	**O[C@@H](CC(=O)c1ccc(Cl)c(Cl)c1)C(=O)[O-]**	**C10 H7 F2 O4**	**6.4**	**420**	
18	26	O[C@H](CC(=O)c1ccc(Cl)c(Cl)c1)C(=O)[O-]	C10 H7 Cl2 O4	4.8	14400	
**19**	**27**	**[O-]C(=O)[C@H](CC(=O)c1ccc(Cl)c(Cl)c1)Cc2ccccc2**	**C17 H13 Cl2 O3**	**5.6**	**2300**	
20	28	[O-]C(=O)[C@@H](CC(=O)c1ccc(Cl)c(Cl)c1)Cc2ccccc2	C10 H5 Cl2 O3	5.0	10700	
**21**	**2a**	**[NH3+][C@H](CC(=O)c1ccc(Cl)c(Cl)c1)C(=O)[O-]**	**C10 H9 Cl2 N O3**	**6.5**	**300**	[6]

**22**	**2b**	**[NH3+][C@@H](CC(=O)c1ccc(Cl)c(Cl)c1)C(=O)[O-]**	**C10 H11 N O3**	**5.7**	**2100**	
**23**	**3a**	**[NH3+][C@H](CC(=O)c1ccccc1)C(=O)[O-]**	**C10 H9 Cl2 N O3**	**4.8**	**16000**	
**24**	**3**	**[NH3+][C@@H](CC(=O)c1cccc(c1)[N+](=O)[O-])C(=O)[O-]**	**C10 H10 N2 O5**	**6.0**	**900**	[7]
**25**	**9**	**[O-]C(=O)[C@H]1C[C@@H]1C(=O)c2ccc(Cl)c(Cl)c2**	**C11 H7 Cl2 O3**	**7.7**	**20**	
26	7	[O-]C(=O)CCn1ncc2ccccc12	C10 H8 Cl N2 O2	5.2	6310	
27	8	[O-]C(=O)CCn1ncc2cc(Cl)ccc12	C11 H10 Cl N2 O2	6.0	1000	
28	9	[O-]C(=O)CCn1ncc2ccc(Cl)cc12	C11 H10 Cl N2 O2	7.3	50	
29	10	C[C@H](CC(=O)[O-])n1ncc2ccc(Cl)cc12	C10 H8 Cl N2 O3	5.3	5012	
30	11	C[C@H](Cn1ncc2ccc(Cl)cc12)C(=O)[O-]	C10 H10 Cl N3 O2	5.6	2512	
31	12	O[C@H](Cn1ncc2ccc(Cl)cc12)C(=O)[O-]	C11 H10 Cl N2 O2	6.2	631	
32	13	[NH3+][C@H](Cn1ncc2ccc(Cl)cc12)C(=O)[O-]	C9 H6 Cl N2 O2	5.6	2512	
33	14	[O-]C(=O)CCCn1ncc2ccc(Cl)cc12	C11 H10 Cl N2 O2	5.5	3162	
34	15	[O-]C(=O)Cn1ncc2ccc(Cl)cc12	C11 H9 Cl N O2	7.0	100	
35	16	Cc1nn(CCC(=O)[O-])c2cc(Cl)ccc12	C9 H7 Cl N3 O2	5.7	1995	
36	17	[O-]C(=O)CCn1ccc2ccc(Cl)cc12	C10 H8 Cl N2 O2	6.9	126	
37	18	[O-]C(=O)CCn1nnc2ccc(Cl)cc12	C10 H8 Cl N2 O3	6.3	501	
38	19	[O-]C(=O)CCn1cnc2ccc(Cl)cc12	C10 H7 Cl N O4	5.0	10000	
39	20	[O-]C(=O)CCN1C(=O)Nc2ccc(Cl)cc12	C10 H7 Cl N O3	5.2	6310	
40	21	[O-]C(=O)CCN1C(=O)Oc2ccc(Cl)cc12	C11 H9 Cl N O4	7.9	13	[8]
41	22	[O-]C(=O)CCc1noc2ccc(Cl)cc12	C11 H7 N2 O4	7.6	25	
42	23	[O-]C(=O)CCN1C(=O)COc2ccc(Cl)cc12	C11 H10 N O5	7.1	79	
43	24	[O-]C(=O)CCN1C(=O)Oc2ccc(cc12)C#N	C11 H7 F3 N O4	6.2	631	
44	25	COc1ccc2OC(=O)N(CCC(=O)[O-])c2c1	C10 H7 Br N O4	6.0	1000	
45	26	[O-]C(=O)CCN1C(=O)Oc2ccc(cc12)C(F)(F)F	C11 H10 N O4	5.3	5012	
46	27	[O-]C(=O)CCN1C(=O)Oc2ccc(Br)cc12	C11 H9 Cl N O4	7.4	40	
47	28	Cc1ccc2OC(=O)N(CCC(=O)[O-])c2c1	C14 H15 Cl N O4	5.5	3162	
48	29	Cc1cc(Cl)cc2N(CCC(=O)[O-])C(=O)Oc12	C11 H9 Cl N O5	7.2	63	
49	30	Cc1cc2OC(=O)N(CCC(=O)[O-])c2cc1Cl	C12 H11 Cl N O5	7.9	13	
50	31	[O-]C(=O)CCN1C(=O)Oc2cc(Cl)c(Cl)cc12	C12 H11 Cl N O4	8.2	6	
**51**	**34**	**CCc1cc2OC(=O)N(CCC(=O)[O-])c2cc1Cl**	**C13 H11 Cl N O5**	**8.0**	**10**	
**52**	**35**	**CC(C)Cc1cc2OC(=O)N(CCC(=O)[O-])c2cc1Cl**	**C11 H9 Cl N O4**	**7.0**	**100**	
**53**	**36**	**COc1cc2OC(=O)N(CCC(=O)[O-])c2cc1Cl**	**C10 H6 Cl2 N O4**	**7.9**	**13**	
**54**	**37**	**CCOc1cc2OC(=O)N(CCC(=O)[O-])c2cc1Cl**	**C11 H6 Cl N2 O2**	**8.3**	**5**	
**55**	**39**	**[O-]C(=O)CCN1C(=O)Oc2cc(OC3CC3)c(Cl)cc12**	**C11 H5 Cl2 N2 O2**	**8.5**	**3**	
56	6	[O-]C(=O)c1cc(ncn1)c2cccc(Cl)c2	C10 H9 N2 O2	9.3	0.5	
57	7	[O-]C(=O)c1cc(ncn1)c2ccc(Cl)c(Cl)c2	C10 H8 Cl N2 O2	9.2	0.6	
58	46	[O-]C(=O)c1cc(ncn1)c2ccccc2	C11 H7 N2 O2	7.4	38	
59	52	[O-]C(=O)c1cc(ncn1)c2ccc(Cl)cc2	C11 H6 Cl N2 O2	8.0	11	
60	53	[O-]C(=O)c1cc(ncn1)c2cc(Cl)cc(Cl)c2	C11 H5 Cl2 N2 O2	7.7	19	
61	54	[O-]C(=O)c1cc(ncn1)c2cccc(F)c2	C11 H6 F N2 O2	9.5	0.3	[3]
62	55	[O-]C(=O)c1cc(ncn1)c2ccc(F)c(Cl)c2	C11 H5 Cl F N2 O2	9.0	0.9	
63	56	[O-]C(=O)c1cc(ncn1)c2ccc(Cl)c(F)c2	C11 H5 Cl F N2 O2	9.5	0.3	
64	57	[O-]C(=O)c1cc(ncn1)c2cccc(Cl)c2F	C11 H5 Cl F N2 O2	9.7	0.2	
65	58	[O-]C(=O)c1cc(ncn1)c2ccc(F)c(F)c2	C11 H5 F2 N2 O2	9.7	0.2	

66	59	[O-]C(=O)c1cc(ncn1)c2ccc(F)cc2F	C11 H5 F2 N2 O2	7.1	76	
67	60	[O-]C(=O)c1cc(ncn1)c2ccc(Cl)cc2F	C11 H5 Cl F N2 O2	7.4	38	
68	61	[O-]C(=O)c1cc(ncn1)c2ccc(c(Cl)c2)C(F)(F)F	C12 H5 Cl F3 N2 O2	8.3	5	
69	62	[O-]C(=O)c1cc(ncn1)c2cccc(c2)C(F)(F)F	C12 H6 F3 N2 O2	7.7	22	
70	63	[O-]C(=O)c1cc(ncn1)c2ccccc2C(F)(F)F	C12 H6 F3 N2 O2	5.2	6510	
71	64	Cc1cccc(c1)c2cc(ncn2)C(=O)[O-]	C12 H9 N2 O2	7.8	17	
72	65	[O-]C(=O)c1cc(ncn1)c2ccc(F)c(c2)C(F)(F)F	C12 H5 F4 N2 O2	7.5	32	
73	66	Cc1ccc(cc1Cl)c2cc(ncn2)C(=O)[O-]	C12 H8 Cl N2 O2	9.2	0.7	
74	67	Cc1ccc(cc1F)c2cc(ncn2)C(=O)[O-]	C12 H8 F N2 O2	9.0	0.9	
75	68	COc1cc(ccc1Cl)c2cc(ncn2)C(=O)[O-]	C12 H8 Cl N2 O3	7.2	60	
76	69	[O-]C(=O)c1cc(ncn1)c2ccc(Cl)c(OCCN3CCOCC3)c2	C17 H17 Cl N3 O4	5.7	2050	
77	70	[O-]C(=O)c1cc(ncn1)c2ccc(OCCN3CCOCC3)c(Cl)c2	C17 H17 Cl N3 O4	7.1	83	
78	71	[O-]C(=O)c1cc(ncn1)c2ccc(N3CCCC3)c(Cl)c2	C15 H13 Cl N3 O2	6.3	550	
79	72	[O-]C(=O)c1cc(ncn1)c2ccc(OC(F)(F)F)c(Cl)c2	C12 H5 Cl F3 N2 O3	8.7	2.1	
80	73	COc1ccc(cc1Cl)c2cc(ncn2)C(=O)[O-]	C12 H8 Cl N2 O3	8.9	1.2	
81	76	[O-]C(=O)c1cc(ncn1)c2ccc(OCC3CC3)c(Cl)c2	C15 H12 Cl N2 O3	7.3	49	
**82**	**8**	**[O-]C(=O)CCN1C(=O)Oc2cc(OCC3CC3)c(Cl)cc12**	**C14 H13 Cl N O5**	**8.0**	**10**	[2]
**83**	**9**	**[O-]C(=O)CCN1C(=O)Oc2cc(OCC3CCC3)c(Cl)cc12**	**C15 H15 Cl N O5**	**7.6**	**25**	

Model 3

№	Comp name	Smiles	Molecular Formula	KMO pIC50	KMO IC50, nM	reference
**1**	**11**	**[O-]C(=O)CCN1C(=O)Oc2cc(OCCN3CCCC3)c(Cl)cc12**	**C16 H18 Cl N2 O5**	**6.1**	**794**	[2]
**2**	**12**	**[O-]C(=O)CCN1C(=O)Oc2cc(OC(=O)c3ccccc3)c(Cl)cc12**	**C17 H11 Cl N O6**	**7.6**	**25.1**	
3	13	[O-]C(=O)CCN1C(=O)Oc2cc(OCc3ccccn3)c(Cl)cc12	C16 H12 Cl N2 O5	8.5	3.16	
**4**	**15**	**C[C@@H](Oc1cc2OC(=O)N(CCC(=O)[O-])c2cc1Cl)c3ccccn3**	**C17 H14 Cl N2 O5**	**8.6**	**2.51**	
**5**	**16**	**C[C@H](Oc1cc2OC(=O)N(CCC(=O)[O-])c2cc1Cl)c3ccccn3**	**C17 H14 Cl N2 O5**	**6.6**	**251**	
**6**	**17**	**C[C@@H](Oc1cc2OC(=O)N(CCC(=O)[O-])c2cc1Cl)c3occn3**	**C15 H12 Cl N2 O6**	**8.5**	**3.16**	
**7**	**18**	**C[C@@H](Oc1cc2OC(=O)N(CCC(=O)[O-])c2cc1Cl)c3ncccn3**	**C16 H13 Cl N3 O5**	**8.3**	**5.01**	
**8**	**19**	**C[C@@H](Oc1cc2OC(=O)N(CCC(=O)[O-])c2cc1Cl)c3cccnn3**	**C16 H13 Cl N3 O5**	**8.9**	**1.26**	
**9**	**20**	**C[C@@H](Oc1cc2OC(=O)N(CCC(=O)[O-])c2cc1Cl)c3cc(C)ccn3**	**C18 H16 Cl N2 O5**	**8.4**	**3.98**	
**10**	**21**	**C[C@@H](Oc1cc2OC(=O)N(CCC(=O)[O-])c2cc1Cl)c3ccc(C)cn3**	**C18 H16 Cl N2 O5**	**8.5**	**3.16**	
**11**	**22**	**C[C@@H](Oc1cc2OC(=O)N(CCC(=O)[O-])c2cc1Cl)c3ccc(Cl)cn3**	**C17 H13 Cl2 N2 O5**	**8.5**	**3.16**	
**12**	**23**	**C[C@@H](Oc1cc2OC(=O)N(CCC(=O)[O-])c2cc1Cl)c3ccc(F)cn3**	**C17 H13 Cl F N2 O5**	**8.6**	**2.51**	
**13**	**24**	**C[C@@H](Oc1cc2OC(=O)N(CCC(=O)[O-])c2cc1Cl)c3cccc(C)n3**	**C18 H16 Cl N2 O5**	**8.3**	**5.01**	
**14**	**25**	**C[C@@H](Oc1cc2OCC(=O)N(CCC(=O)[O-])c2cc1Cl)c3ccccn3**	**C18 H16 Cl N2 O5**	**8.3**	**5.01**	
15	26	C[C@@H](Oc1cc2ccn(CCC(=O)[O-])c2cc1Cl)c3ccccn3	C18 H16 Cl N2 O3	8.3	5.01	
16	27	C[C@@H](Oc1cc2cnn(CCC(=O)[O-])c2cc1Cl)c3ccccn3	C17 H15 Cl N3 O3	8.5	3.16	
17	28	C[C@@H](Oc1cc2onc(CCC(=O)[O-])c2cc1Cl)c3ccccn3	C17 H14 Cl N2 O4	8.3	5.01	
**18**	**29**	**C[C@@H](Oc1cc2SC(=O)N(CCC(=O)[O-])c2cc1Cl)c3ccccn3**	**C17 H14 Cl N2 O4 S**	**8.7**	**2.00**	
19	30	C[C@@H](Oc1cc2onc(CCC(=O)[O-])c2cc1Cl)c3ccc(C)cn3	C18 H16 Cl N2 O4	8.5	3.16	
20	31	C[C@@H](Oc1cc2onc(CCC(=O)[O-])c2cc1Cl)c3ccc(Cl)cn3	C17 H13 Cl2 N2 O4	8.5	3.16	
21	32	C[C@@H](Oc1cc2onc(CCC(=O)[O-])c2cc1Cl)c3ccc(F)cn3	C17 H13 Cl F N2 O4	8.5	3.16	
22	33	C[C@@H](Oc1cc2onc(CCC(=O)[O-])c2cc1Cl)c3ccc(C)nn3	C17 H15 Cl N3 O4	8.7	2.00	

* All of the listed compounds were used to validate the protein-ligand complex pharmacophores, as well as served as the initial input molecules for DUD-E decoy generation. Compound highlighted in bold were included in the docking accuracy validation test.

Supplementary References

1. Röver, S.; Cesura, A.M.; Huguenin, P.; Kettler, R.; Szente, A. Synthesis and Biochemical Evaluation of N-(4-Phenylthiazol-2-yl)benzenesulfonamides as High-Affinity Inhibitors of Kynurenine 3-Hydroxylase. *J. Med. Chem.* **1997**, *40*, 4378–4385, doi:10.1021/jm970467t
2. Walker, A.L.; Ancellin, N.; Beaufils, B.; Bergeal, M.; Binnie, M.; Bouillot, A.; Clapham, D.; Denis, A.; Haslam, C.P.; Holmes, D.S.; et al. Development of a Series of Kynurenine 3-Monooxygenase Inhibitors Leading to a Clinical Candidate for the Treatment of Acute Pancreatitis. *J. Med. Chem.* **2017**, *60*, 3383–3404, doi:10.1021/acs.jmedchem.7b00055.
3. Toledo-Sherman, L.M.; Prime, M.E.; Mrzljak, L.; Beconi, M.G.; Beresford, A.; Brookfield, F.A.; Brown, C.J.; Cardaun, I.; Courtney, S.M.; Dijkman, U.; et al. Development of a series of aryl pyrimidine kynurenine monooxygenase inhibitors as potential therapeutic agents for the treatment of Huntingtons disease. *J. Med. Chem.* **2015**, *58*, 1159–1183, doi:10.1021/jm501350y.
4. Wilkinson, M. Structural Dynamics and Ligand Binding in Kynurenine-3- monooxygenase, Doctoral Thesis, The University Of Edinburgh, Edinburgh, Scotland 2013.
5. Giordani, A.; Pevarello, P.; Cini, M.; Bormetti, R.; Greco, F.; Toma, S.; Speciale, C.; Varasi, M. 4-Phenyl-4-oxo-butanoic acid derivatives inhibitors of kynurenine 3-hydroxylase. *Bioorg. Med. Chem. Lett.* **1998**, *8*, 2907–2912, doi:10.1016/S0960-894X(98)00517-4.
6. Giordani, A.; Corti, L.; Cini, M.; Bormetti, R.; Marconi, M.; Veneroni, O.; Speciale, C.; Varasi, M. Enantiospecific Synthesis and in vitro Activity of Selective Inhibitors of Rat Brain Kynureninase and Kynurenine-3-Hydroxylase. In *Recent Advances in Tryptophan Research. Advances in Experimental Medicine and Biology*; Springer, Boston, MA, 1996; pp. 531–534.
7. Phillips, R.S.; Iradukunda, E.C.; Hughes, T.; Phillip Bowen, J. Modulation of enzyme activity in the kynurenine pathway by kynurenine monooxygenase inhibition. *Front. Mol. Biosci.* **2019**, *6*, doi:10.3389/fmolb.2019.00003.
8. Liddle, J.; Beaufils, B.; Binnie, M.; Bouillot, A.; Denis, A.A.; Hann, M.M.; Haslam, C.P.; Holmes, D.S.; Hutchinson, J.P.; Kranz, M.; et al. The discovery of potent and selective kynurenine 3-monooxygenase inhibitors for the treatment of acute pancreatitis. *Bioorganic Med. Chem. Lett.* **2017**, *27*, 2023–2028, doi:10.1016/j.bmcl.2017.02.078.
